# Human Intraocular Filariasis Caused by *Pelecitus* sp. Nematode, Brazil

**DOI:** 10.3201/eid1705.101309

**Published:** 2011-05

**Authors:** Odile Bain, Domenico Otranto, Daniel G. Diniz, Jeannie Nascimento dos Santos, Norimar Pinto de Oliveira, Izabela Negrão Frota de Almeida, Rafael Negrão Frota de Almeida, Luciana Negrão Frota de Almeida, Filipe Dantas-Torres, Edmundo Frota de Almeida Sobrinho

**Affiliations:** Author affiliations: Muséum National d’Histoire Naturelle, Paris, France (O. Bain);; Università degli Studi di Bari, Valenzano, Italy (D. Otranto, F. Dantas-Torres);; Universidade Federal do Pará, Belém, Brazil (D.G. Diniz, J. Nascimento dos Santos, I.N. Frota de Almeida, R.N. Frota de Almeida, L.N. Frota de Almeida, E. Frota de Almeida Sobrinho);; Instituto de Medicina do Olho de Tucuruí, Tucuruí, Brazil (N. Pinto de Oliveira)

**Keywords:** Pelecitus sp., nematode, intraocular filariasis, zoonosis, human, eye, parasites, Loaina, Brazil, dispatch

## Abstract

A male nematode was extracted from iris fibers of a man from the Brazilian Amazon region. This nematode belonged to the genus *Pelecitus* but was distinct from the 16 known species in this genus. Similarities with *Pelecitus* spp. from neotropical birds suggested an avian origin for this species.

Filarial nematodes have been found in the eyes and periorbital region of humans worldwide (*1**–**4*). However, rarely have the worms been removed and morphologically described. The main human filarial parasites are *Wuchereria bancrofti* and *Brugia malayi*, whose adults live in the lymphatic system, and *Loa loa*, which infects subcutaneous tissues. In addition, some filarioids have an animal origin, either from domestic mammals, such as for *Dirofilaria* spp., or from wild mammals, including *Onchocerca*, *Molinema*, and *Loaina* spp (*1**,**2**,**5*).

Nematode identification at the species level might be supported by anamnestic information, such as host and geographic location. However, for a reliable, definitive, species identification, proper morphologic or molecular diagnosis is needed. Clinical reports may provide a useful database for better understanding of the zoonotic potential of little-known filarioids infecting wild animals. We report a case of human intraocular filariasis caused by a *Pelecitus* sp., briefly describe the main morphologic features for nematode identification, and suggest the origin of this zoonotic infection.

## The Study

On August 2007, a 29-year-old man from Tucuruí in northern Brazil, who worked in power grid maintenance in a forested area, came to his ophthalmologist with an intraocular larva in the left eye. There was no familial history of ophthalmologic disorders, and ophthalmologic examinations showed that the patient had visual acuity and corrected vision of 20/25 in both eyes. Biomicroscopy showed a transparent cornea in the right eye without lesions or edema, an anterior cavity without an inflammatory reaction, and an anterior subcapsular cataract of +/4+. The cornea in the left eye was transparent and did not have lesions or edema, and the anterior chamber did not show an inflammatory reaction. No funduscopic alterations were found in either eye by direct and indirect ophthalmoscopic examination.

**Figure 1:**
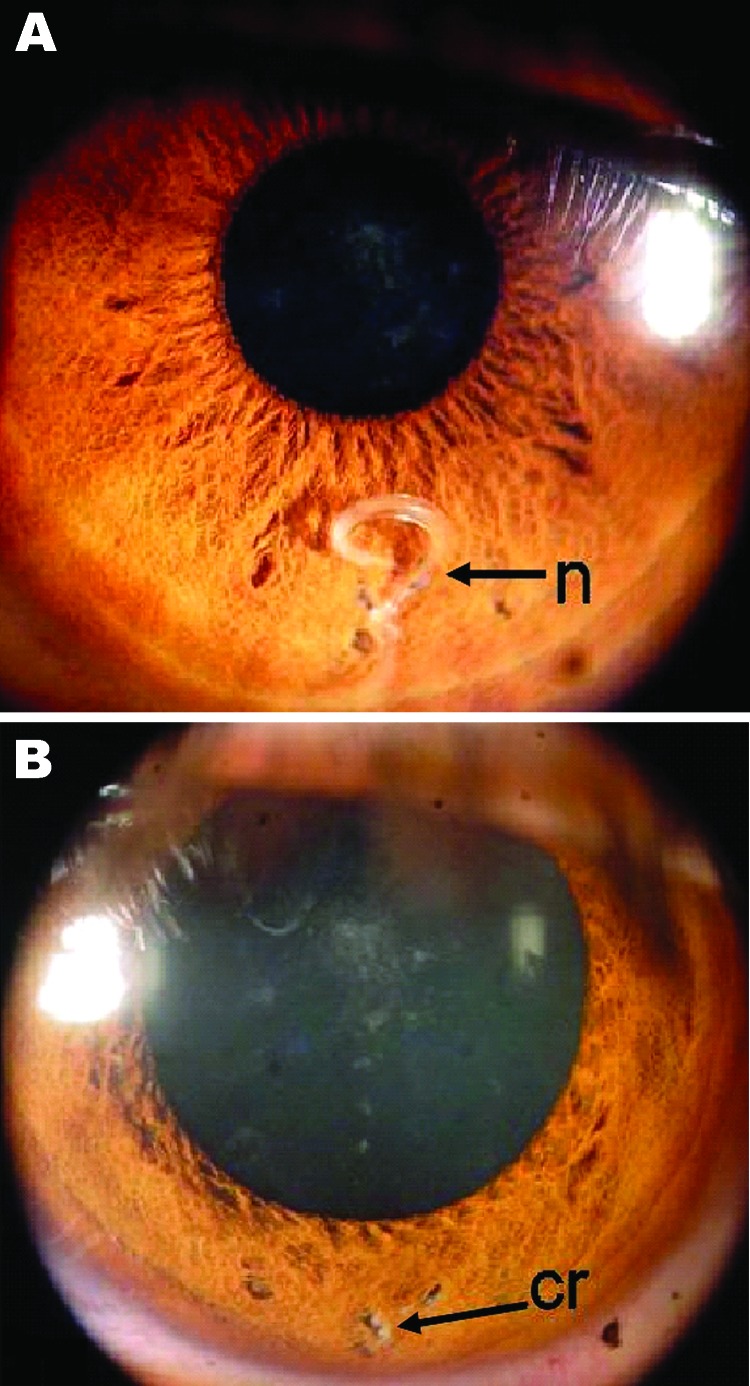
Eye of the patient, a 29-year-old man from Brazil. A) Nematode (n) between muscle fibers of the iris. B) Iris after surgery, showing a mild residual scar (cr) in the region where the nematode had been located.

An ≈4-mm worm with undulating movements was observed between the muscular fibers of the iris (Figure 1, panel A). The patient underwent surgery 1 day after the consultation, and he consented to the publication of this clinical case. After peribulbar anesthesia, a 2-mm corneal incision was made at the 11 o’clock position. The nematode was extracted by aspiration (Video) and placed in saline solution. No surgical complications occurred (Figure 1, panel B), and the patient did not have ocular symptoms during the 6 months after surgery.

**Video:**
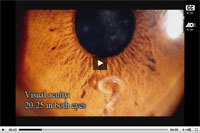
Surgical removal of a *Pelecitus* sp. nematode from the left eye of a 29-year-old man, Brazil. A portion of the material in this video was previously published in the journal Parasites and Vectors (http://www.parasitesandvectors.com/content/pdf/1756-3305-4-41.pdf).

Direct Link: http://streaming.cdc.gov/vod.php?id=bec4b66c4804f440dce5072b04eeba0c20110426172737138

**Figure 2:**
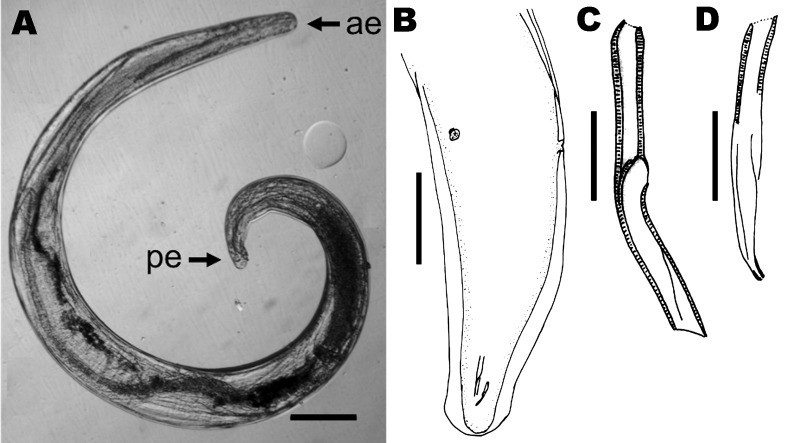
Parasitic nematode isolated from the eye of the patient, a 29-year-old man from Brazil. A) Nematode that was removed from the iris, showing anterior (ae) and posterior (pe) extremities. Scale bar = 200 µm. B) Caudal region, subdorsal view, showing lateral alae, spicules, and the 2 postdeirids. Scale bar = 150 µm. C) Left spicule; D) right spicule. Scale bars = 20 µm.

The worm was fixed in 2% acetic acid, 3% formaldehyde, and 95% ethanol; mounted in glycerine jelly; and later transferred into lactophenol. The specimen was preserved in absolute alcohol at the National Museum of Natural History (Paris, France) (accession no. 138 YU). This male nematode (length 4.5 mm, width 300 µm at mid-body) had a coiled and twisted body that tapered at both extremities (Figure 2, panel A). The cuticle (thickness 6 µm) showed 2 rounded, lateral, cuticular alae (thickness 20 μm) along the body and postdeirids 530 µm from the posterior extremity (Figure 2, panel B). The head was bluntly rounded and contained 4 externolabial papillae, 4 cephalic papillae, 2 amphids, and a buccal cavity (length 5 µm, width 4.5 µm) with a tiny cuticular ring. The nerve ring was 165 µm from the anterior end. The esophagus was 765 µm long, increased slightly in diameter in the posterior half, and did not have a distinct glandular part. The large caudal alae had 2 granular inclusions on each lateral side. The tail was 48 µm long. Five pairs of caudal papillae (2 pedunculated, precloacal, lateral; 1 small subventral closely posterior to the cloacal opening; and 2 pedunculated lateral pairs on posterior half of the tail) were observed, and the 2 phasmids were subterminal. The 2 spicules (length 66 µm and 82 µm) (Figure 2, panels C, D) were dissimilar. The larger left spicule had a typical beveled extremity.

Many morphologic characteristics of the filarial worm resembled those of *Pelecitus* spp (*6*). (coiled and twisted body that was attenuated at both extremities; lateral alae from the cervical region to distal tip of body; postdierids within alae in the posterior half of body; and a delicate, preesophageal, cuticular ring). *Pelecitus* spp. include mainly parasites of birds and a few mammals, some of which have been identified as *Loaina* spp (*6**,**7*).

The specimen from the patient was compared with the 16 known species of *Pelecitus* described (*6**,**8**,**9*), but the specimen did not match any of them. The worm differed from the only 2 species found in lagomorphs (*P. scapiceps* and *P. meridionaleporinus*) (*9*) in North America and Mexico, which had a beveled extremity on the right spicule (*8**,**9*) instead of the left spicule.

A small male filaria was recovered from the anterior chamber of a human eye in Colombia (*5*). This filaria was originally assigned to the genus *Loaina* but was later identified as a species of *Pelecitus* (*6*). Like the nematode specimen we describe, it was morphologically similar to some species that infect birds.

## Conclusions

We assigned the worm found in the anterior chamber of the eye of the patient to the genus *Pelecitus* (*6*). The species of *Pelecitus* that infected the eyes of 2 humans (reported here and in Colombia) (*5*) remains unidentified. These cases were found in the tropical Amazon region (Pará, Brazil) and the Department of Antioquia (northwestern Colombia). The male specimen of *Pelecitus* sp. described here and the species that infected a human in Colombia are similar but distinct. However, in both cases, a mammalian origin of these zoonotic agents seems unlikely because of differences identified by comparing these worms with parasitic species infecting lagomorphs (*8**,**9*). Both cases of human infection with *Pelecitus* spp. more likely have an avian origin. Vectors of *Pelecitus* spp. are mosquitoes, chewing lice, and tabanids, as shown with the 3 cycles elucidated (*10**–**12*).

Although infection of birds in South America by *Pelecitus* spp. has been reported (*13**,**14*), information on this taxon is scant. Many nematode species have not been identified because of lack of basic information on filarial fauna of animals. This dearth of information is particularly true for regions, such as the Amazon rain forest in Brazil, where wide biodiversity and many unidentified animal and plant species are found (*15*). Consequently, species identification of filarioid nematodes that infect human eyes is difficult if not impossible. However, our identification of this filarid should help clarify the zoonotic role of filarioid infections in humans in tropical regions and increase awareness of physicians and ophthalmologists of the variety of nematodes that may be found in the human eye.

## References

[1] Beaver PC. Intraocular filariasis: a brief review. Am J Trop Med Hyg. 1989;40:40–5. 2644857 10.4269/ajtmh.1989.40.40

[2] Orihel TC, Eberhard ML. Zoonotic filariasis. Clin Microbiol Rev. 1998;11:366–81. 9564568 10.1128/cmr.11.2.366 PMC106837

[3] Sallo F, Eberhard ML, Fok E, Baska F, Hatvani I. Zoonotic intravitreal *Onchocerca* in Hungary. Ophthalmology. 2005;112:502–4. 10.1016/j.ophtha.2004.10.036 15745781

[4] Pampiglione S, Rivasi F, Gustinelli A. Dirofilarial human cases in the Old World, attributed to *Dirofilaria immitis*: a critical analysis. Histopathology. 2009;54:192–204. 10.1111/j.1365-2559.2008.03197_a.x 19207944

[5] Botero D, Aguledo LM, Uribe FU, Esslinger JH, Beaver PC. Intraocular filaria, a *Loaina* species, from man in Colombia. Am J Trop Med Hyg. 1984;33:578–82. 6476200 10.4269/ajtmh.1984.33.578

[6] Bartlett CM, Greiner EC. A revision of *Pelecitus* Railliet & Henry, 1910 (Filarioidea, Dirofilariinae) and evidence for the “capture” by mammals of filarioids from birds. Bulletin du Muséum National d’Histoire Naturelle (Paris). 1986;8:47–99.

[7] Eberhard ML, Orihel TC. *Loaina* gen. n. (Filarioidea: Onchocercidae) for the filariae parasitic in rabbits in North America. Proceedings of the Helminthological Society of Washington. 1984;51:49–53.

[8] Bartlett CM. Zoogeography and taxonomy of *Dirofilaria scapiceps* (Leidy, 1886) and *D. uniformis* Price, 1957 (Nematoda: Filarioidea) of lagomorphs in North America. Can J Zool. 1983;61:1011–22. 10.1139/z83-135

[9] Jiménez-Ruiz FA, Gardner SL, Cervantes FA, Lorenzo C. A new species of *Pelecitus* (Filarioidea: Onchocercidae) from the endangered Tehuantepec jackrabbit *Lepus flavigularis.* J Parasitol. 2004;90:803–7. 10.1645/GE-213R1 15357073

[10] Bartlett CM. Development of *Dirofilaria scapiceps* (Leidy, 1886) (Nematoda: Filarioidea) in *Aedes* spp. and *Mansonia perturbans* (Walker) and responses of mosquitoes to infection. Can J Zool. 1984;62:112–29. 10.1139/z84-019

[11] Bartlett CM, Anderson RC. *Pelecitus fulicaeatrae* (Nematoda: Filarioidea) of coots (Gruiformes) and grebes (Podicipediformes): skin-inhabiting microfilariae and development in Mallophaga. Can J Zool. 1987;65:2803–12. 10.1139/z87-423

[12] Spratt DM. Natural occurrence, histopathology and developmental stages of *Dirofilaria roemeri* in the intermediate host. Int J Parasitol. 1972;2:201–8. 10.1016/0020-7519(72)90007-0 4652607

[13] Pinto RM, Vicente JJ, Noronha D. Nematode parasites of Brazilian psittacid birds, with emphasis on the genus *Pelecitus* Railliet & Henry, 1910. Mem Inst Oswaldo Cruz. 1993;88:279–84. 10.1590/S0074-02761993000200016

[14] Oniki Y, Kinsella JM, Willis EO. *Pelecitus helicinus* Railliet & Henry, 1910 (Filarioidea, Dirofilariinae) and other nematode parasites of Brazilian birds. Mem Inst Oswaldo Cruz. 2002;97:597–8. 10.1590/S0074-02762002000400027 12118298

[15] Barlow J, Gardner TA, Araujo IS, Avila-Pires TC, Bonaldo AB, Costa JE, Quantifying the biodiversity value of tropical primary, secondary, and plantation forests. Proc Natl Acad Sci U S A. 2007;104:18555–60. 10.1073/pnas.0703333104 18003934 PMC2141815

